# Incidence, presentation and outcome of acute aortic dissection: results from a population-based study

**DOI:** 10.1136/openhrt-2023-002595

**Published:** 2024-03-13

**Authors:** Christian Smedberg, Rebecka Hultgren, Christian Olsson, Johnny Steuer

**Affiliations:** 1 Department of Clinical Science and Education Södersjukhuset, Karolinska Institutet, Stockholm, Sweden; 2 Department of Surgery, Södersjukhuset AB, Stockholm, Sweden; 3 Department of Molecular Medicine and Surgery, Karolinska Institutet, Stockholm, Sweden; 4 Department of Vascular Surgery, Karolinska University Hospital, Stockholm, Sweden; 5 Department of Cardiothoracic Surgery, Karolinska University Hospital, Stockholm, Sweden

**Keywords:** Aneurysm, Dissecting, Biomarkers, Endovascular Procedures

## Abstract

**Objectives:**

To describe the incidence of acute aortic dissection in a clearly defined population, to assess onset symptoms and admission biochemical marker levels and to analyse variables potentially associated to mortality.

**Methods:**

Medical records and CT angiograms of all patients hospitalised for acute aortic dissection in the Stockholm County during the 5-year period 2012–2016 were reviewed. The patients were followed until date of death or until 31 December 2020. The annual incidence was determined. Associations between clinical and biochemical variables and 30-day mortality, respectively, were analysed using multivariable logistic regression models.

**Results:**

A total of 344 patients were included. The mean annual incidence of acute aortic dissection was 4.1 per 100 000. Median age was 67 years (range 24–91) and 34% (n=118) were women. Type A dissection was predominant; 220 patients (64%) had type A and 124 (36%) had type B. Painless dissection was more common in type A than in type B (18% vs 15%, p=0.003). Type A dissection patients also more commonly had elevated plasma troponin T (44% vs 21%, p<0.001) and thrombocytopenia (26% vs 15%, p=0.010) than type B dissection patients on admission. Overall, 30-day mortality was 28% in type A and 11% in type B (p<0.001). Both painless dissection (OR 4.30, 95% CI 1.80 to 10.28, p=0.001) and elevated troponin T (OR 3.78, 95% CI 2.01 to 7.12, p<0.001), respectively, were associated with increased 30-day mortality in all acute aortic dissection patients. Thrombocytopenia was associated with elevated 30-day mortality only in patients with type A (OR 3.09, 95% CI 1.53 to 6.21, p=0.002).

**Conclusions:**

Nearly two-thirds of acute aortic dissection patients had type A. Levels of troponin T and platelets, respectively, paired with presence or absence of typical symptoms may become useful adjuncts in risk stratification of patients with acute aortic dissection.

**WHAT IS ALREADY KNOWN ON THIS TOPIC**

- From the few population-based studies that exist on aortic dissection (AD), annual incidence of 2.5–7.1 per 100 000 has been reported. Atypical presentation without pain has been described to be uncommon but associated with worse outcome. Elevation of troponin may coincide with acute AD and might delay diagnosis.

**WHAT THIS STUDY ADDS**

- The annual incidence of acute AD in Stockholm County was 4.1 per 100 000. Painless dissection was more frequent than previously described and it was associated with increased early mortality. Furthermore, elevation of plasma troponin T on admission was more common in patients with type A than type B acute AD and associated with higher early mortality in both patient groups.

**HOW THIS STUDY MIGHT AFFECT RESEARCH, PRACTICE OR POLICY**

- As acute AD may present without pain and with elevation of troponin T, it should be considered as differential diagnosis in patients with atypical acute symptoms of severe disease or in acute onset chest pain without coronary ischaemia since early diagnosis of acute AD is of utmost importance to improve the outcome.

## Introduction

Acute aortic dissection (AD) is one of the potentially most lethal cardiovascular emergencies unless diagnosed early with initiation of adequate management. In less than half of the patients, the condition is suspected at the first evaluation.^1^ Data from a Swedish nationwide retrospective register study demonstrated a mean annual incidence of acute AD of 7.2 per 100 000.^2^

The most established classification schemes, the Stanford anatomical classification differentiating between type A dissection (TAD) and type B dissection (TBD), respectively, and the temporal distinction, defining acute as 14 days or less from onset, are still valid.^3 4^ Nonetheless, modern treatment strategies are emerging, necessitating a more precise and in-depth categorisation.

The majority of patients with acute AD present with either chest pain or back pain or both.^5–8^ Still, some patients present with atypical symptoms and are hospitalised for other predominant symptoms. This type of onset of AD is often designated painless dissection, which, according to the International Registry of Acute Aortic Dissection (IRAD), occurs in 6% of AD patients.^9^ In such patients, the diagnosis may be delayed with ensuing worse outcome.^5 7 9 10^

The role of biochemical markers or laboratory workup in the early management of patients with AD is limited in daily clinical practice. D-dimer is the only marker shown to be of some practical clinical benefit as it has high sensitivity for the detection of AD.^11 12^ A small number of previous reports have indicated that there might be an association between elevated admission troponin level as well as thrombocytopenia, respectively, and increased early mortality in acute AD.^13 14^

The aim was to describe the incidence of acute AD in a clearly defined population, to characterise symptoms and describe levels of biochemical markers at onset and, finally, to analyse variables potentially associated with mortality.

## Methods

### Study design and setting

This was a population-based retrospective cohort study, including all patients hospitalised with acute AD (code I71.0, International Classification of Diseases, 10th revision (ICD-10)) in the County of Stockholm, with a well-defined catchment area, during the 5-year period 1 January 2012 through 31 December 2016. The patients were followed until date of death or until 31 December 2020. The Stockholm County is the largest county in Sweden. It had 2.3 million inhabitants in 2016. Of six regional hospitals, vascular surgery is centralised to two, the Karolinska University Hospital and the Stockholm South Hospital. Cardiothoracic surgery is only carried out at the Karolinska University Hospital. By way of the unique Swedish personal identity number, follow-up could be completed in all patients.

### Data collection

The patients were identified through local hospital registers. Patients with traumatic, iatrogenic or chronic AD were excluded. Demographic data, onset symptoms, biochemical marker levels and intraoperative variables were extracted from medical records. Patients presenting with atypical symptoms, not describing pain on admission, were categorised as having painless dissection. Data on open surgical repair (OSR) and on endovascular repair, including Thoracic EndoVascular Aortic Repair (TEVAR) and abdominal EVAR were registered. Dates of death were documented and 30-day, 1-year and 3-year mortality data were analysed.

Patients with AD involving the ascending aorta were categorised as TAD and the remaining patients as TBD. CT angiograms were retrospectively reviewed to verify the classification into TAD and TBD, respectively. In TBD patients, the acute phase was defined as the first 14 days from onset of disease. TBD was further classified as complicated or uncomplicated. Complications included rupture/imminent rupture, malperfusion, rapid expansion or aneurysm development. Imminent rupture was defined as periaortic haematoma/inflammation/oedema.

Biochemical marker levels on admission and during the hospital stay were documented. The assays used with corresponding cut-off levels and metric units are described in online supplemental table 1.

10.1136/openhrt-2023-002595.supp1 Supplementary data

The Strengthening the Reporting of Observational Studies in Epidemiology (STROBE) Statement was used in the preparation of the analysis plan and of the manuscript.^15^

### Statistical analysis

Categorical variables were presented with numbers and percentages. Continuous variables were presented with median and range. Differences in categorical variables were analysed using χ^2^ test and differences in continuous variables were analysed with Mann-Whitney U test. Receiver operating characteristic (ROC) curves were produced for admission levels of troponin T and the ability to predict 30-day mortality. Symptoms at onset and levels of biochemical markers on admission, respectively, and the associations with 30-day mortality were analysed using multivariable logistic regression models adjusting for age and sex. Subgroup analyses were performed for TAD and TBD, respectively, as well as for patients with TAD treated with OSR and for patients with acute complicated TBD treated with TEVAR, respectively. Results were presented with ORs with 95% CIs. Mid-term mortality was analysed using Cox proportional hazards models. Results were presented with HRs with 95% CIs. A p value of <0.05 was considered statistically significant for all models. Patients with missing values for biochemical markers were excluded from further analyses for that specific marker. Statistical analyses were carried out using SPSS 28.0 for Windows.

## Results

### Patient characteristics

**Figure 1:**
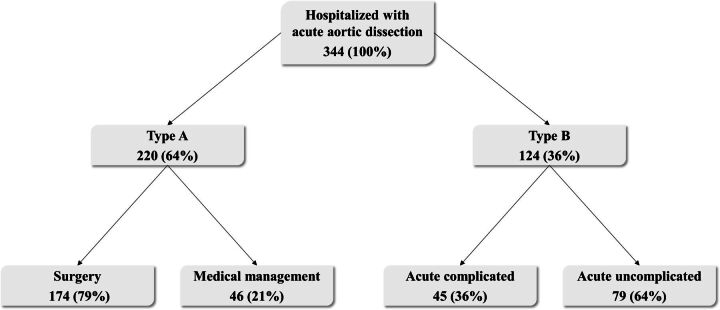
Flowchart of patients hospitalised with aortic dissection in Stockholm County 2012–2016. Patients are subdivided into Stanford type A dissection (TAD) and Stanford type B dissection (TBD), respectively.

During the study period, 344 patients were hospitalised with acute AD (figure 1). Mean age was 67 years (range 24–91) and 34% (n=118/344) were women. Almost two-thirds of the patients had TAD (64%, n=220) and the remaining patients had TBD (36%, n=124) (figure 1). Patients with TBD were older (69 vs 66 years, p=0.036) and had more chronic obstructive pulmonary disease (12% vs 4%, p=0.005) than patients with TAD (table 1). Of patients with TAD, 75% had DeBakey type I extension, whereas in 25% it was limited to the ascending aorta, DeBakey type II. Correspondingly, 24% of cases with TBD were confined to the descending aorta, DeBakey type IIIA, whereas 76% propagated further distally below the diaphragm, DeBakey type IIIB. Mean annual incidence of acute AD in Stockholm County was 4.1 per 100 000, 5.5 per 100 000 in men and 2.8 per 100 000 in women.

**Table 1:** Demographics and pharmacological treatment on admission in patients with acute aortic dissection in Stockholm County 2012–2016

Variable	Total (n=344)	Type A (n=220)	Type B (n=124)	P value
Demographics				
Age (median, range)	67 (24–91)	66 (27–91)	69 (24–91)	0.038
Women	118 (34%)	76 (35%)	42 (34%)	0.899
BMI (kg/m^2^) (SD)	26.5 (4.8)	26.6 (4.4)	26.3 (5.5)	0.266
Hypertension	199 (58%)	121 (55%)	78 (63%)	0.154
Ischaemic heart disease	48 (14%)	33 (15%)	15 (12%)	0.456
AF	38 (11%)	25 (12%)	13 (11%)	0.781
Diabetes	14 (4%)	8 (4%)	6 (5%)	0.588
Kidney failure	12 (4%)	8 (4%)	4 (3%)	0.842
COPD	24 (7%)	9 (4%)	15 (12%)	0.005
Marfan syndrome	7 (2%)	2 (1%)	5 (4%)	0.049
Other connective tissue disorder	6 (2%)	6 (3%)	0	0.064
Smoker	73 (26%)	43 (25%)	30 (28%)	0.438
Former smoker	68 (24%)	45 (26%)	23 (21%)	0.438
Pharmacological agents				
Antiplatelet agent	56 (16%)	30 (14%)	26 (22%)	0.061
Anticoagulant	42 (12%)	23 (11%)	19 (16%)	0.168
Statin	56 (17%)	36 (16%)	20 (17%)	0.957
Antihypertensive agent	173 (51%)	107 (49%)	66 (55%)	0.298
Betablocker	103 (31%)	61 (29%)	42 (36%)	0.156
Angiotensin 2 receptor blocker	58 (17%)	35 (16%)	23 (20%)	0.437
ACE inhibitor	69 (21%)	42 (19%)	27 (23%)	0.423
Calcium channel blocker	58 (17%)	37 (17%)	21 (18%)	0.863

Categorical variables are presented with numbers and percentages. Differences between the type A and type B dissection groups with respect to categorical variables were analysed using χ^2^ test and differences in continuous variables were analysed with Mann-Whitney U test. A p value below 0.05 was considered statistically significant.

AF, atrial fibrillation; BMI, body mass index; COPD, chronic obstructive pulmonary disorder.

### Presenting symptoms

The majority of the patients presented with chest pain as the predominant symptom (61%, n=209), whereas 14% (n=48) had painless dissection. Painless dissection was more common in patients with TAD than in TBD (table 2A). The most common clinical presentation in patients with painless dissection was neurological deficit (33%, n=16/48) (table 2B).

**Table 2:** Onset symptoms in all patients hospitalised with aortic dissection in Stockholm County 2012–2016 (A) and presenting symptoms in patients with painless dissection (B)

(A) variable	Total (n=344)	Type A (n=220)	Type B (n=124)	P value
Chest pain	209 (61%)	152 (69%)	57 (46%)	<0.001
Back pain	56 (16%)	21 (10%)	35 (28%)	<0.001
Abdominal pain	31 (9%)	7 (3%)	24 (19%)	<0.001
Painless	48 (14%)	40 (18%)	8 (7%)	0.003

(B) Variable	Total (n=48)	Type A (n=40)	Type B (n=8)	P value
Cardiac arrest	4 (8%)	3 (7%)	1 (12.5%)	0.640
Unconsciousness	6 (13%)	6 (15%)	–	0.242
Syncope	6 (13%)	6 (15%)	–	0.571
Neurological deficit	16 (33%)	14 (35%)	2 (25%)	0.584
Limb ischaemia	4 (8%)	3 (7%)	1 (12.5%)	0.640
Dyspnoea	9 (19%)	6 (15%)	3 (28.5%)	0.242
Palpitations	2 (4%)	1 (3%)	1 (12.5%)	0.196
Headache	1 (2%)	1 (3%)	–	0.647

Categorical variables are presented with numbers and percentages. Differences between type A and type B dissection with respect to categorical variables were analysed with χ^2^ test, a p value below 0.05 was considered statistically significant.

### Biochemical markers

**Figure 2:**
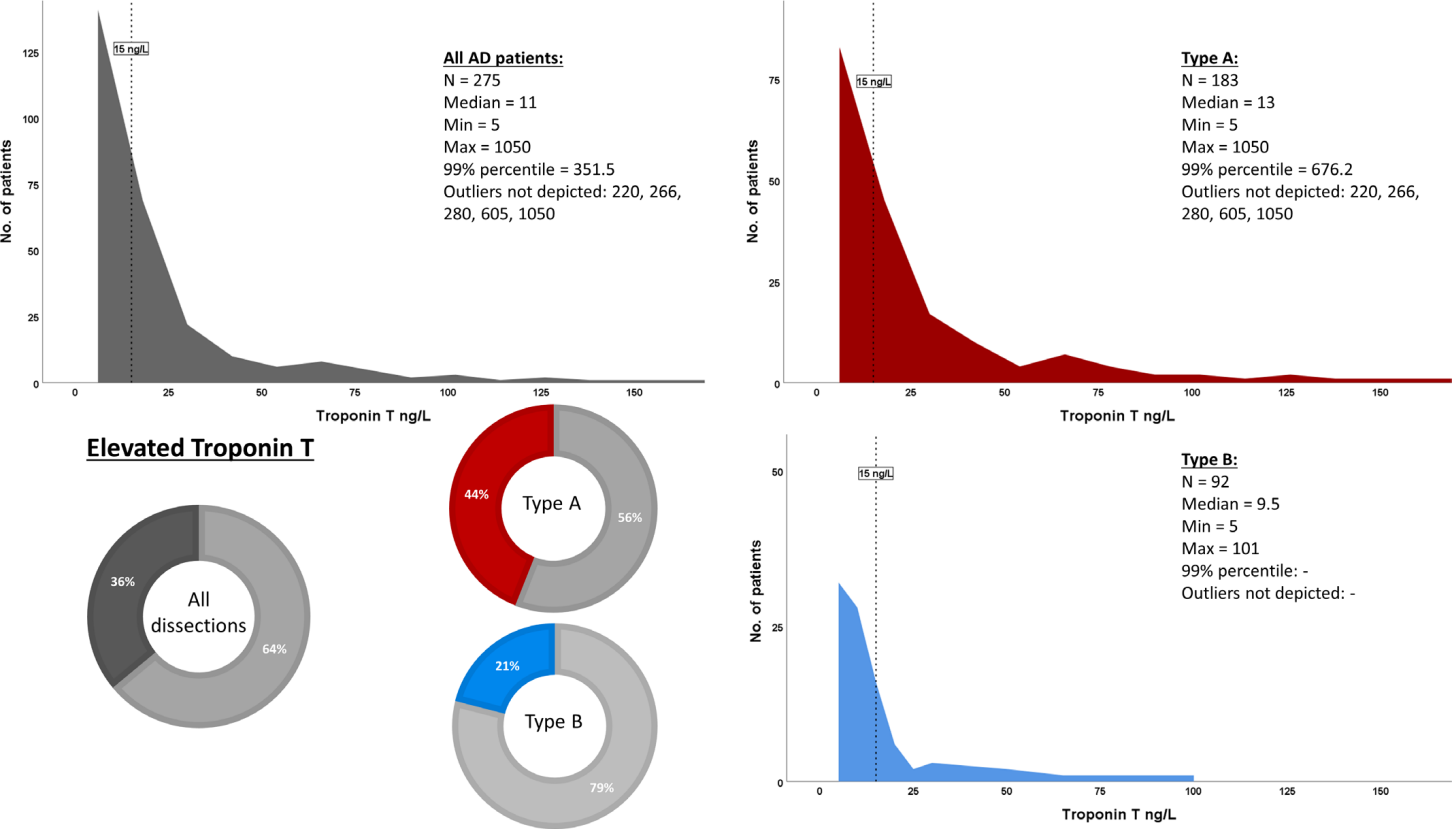
Distribution of levels of troponin T on admission described with histograms. Outliers, troponin T values above 150 ng/L, are not depicted. The proportions of patients with elevated troponin T on admission, defined as >14 ng/L (upper reference level), are depicted as coloured sectors of the circle diagrams. The results are presented for the whole cohort of patients (grey) as well as with the patients subdivided into type A (red) and type B dissection (blue), respectively. AD, aortic dissection.

Biochemical marker levels on admission and during hospitalisation are described in table 3. Admission level of plasma troponin T was analysed in 275 of the 344 patients (80%) with acute AD. In 26% of the patients eventually found to have TBD (n=32/124) and in 17% of the TAD patients (n=37/220), respectively, plasma troponin T quantification had not been requested by the emergency room physician. Median level of troponin T on admission was 11.0 ng/L. The troponin T plasma concentration was 27 ng/L at the 80th percentile, 59 ng/L at the 90th percentile, 87 ng/L at the 95th percentile and 352 ng/L at the 99th percentile, respectively, among all patients with acute AD. Troponin T was elevated (>14 ng/L) in 36% (n=100/275) of the cases on admission, more commonly in patients with TAD than in patients with TBD (44% vs 21%, p<0.001) (table 3, figure 2). ROC curves depicting troponin T level in relation to 30-day mortality demonstrated an area under the curve (AUC) of 0.667 and best discriminatory value of 12.5 (sensitivity 0.74 and 1-specificity of 0.39) for the entire cohort. For TAD separately, the AUC was 0.629 and the best discriminatory value was 12.5 (sensitivity 0.75 and 1-specificity of 0.43) (online supplemental figure 1).

10.1136/openhrt-2023-002595.supp2 Supplementary data

10.1136/openhrt-2023-002595.supp3 Supplementary data

**Table 3:** Levels of biochemical markers in patients with acute aortic dissection in Stockholm County 2012–2016

Variable	Total (n=344)	Type A (n=220)	Type B (n=124)	P value
Troponin T (ng/L) (n=275)				
Level on admission (ng/L)	11 (5–1050)	13 (5–1050)	9.5 (5-101)	0.001
Elevated on admission (n, %)	100 (36%)	81 (44%)	19 (21%)	<0.001
Platelet count (×10^9^) (n=337)				
Level on admission (×10^9^)	192 (59–591)	188 (59–509)	203 (63–591)	0.007
Admission thrombocytopenia (n, %)	74 (22%)	56 (26%)	18 (15%)	0.019
CRP (mg/L) (n=325)				
Level on admission (mg/L)	4 (1-257)	3 (1-257)	5 (1-164)	0.022
Maximum during hospitalisation (mg/L)	205 (1–560)	246 (1–560)	138 (1–456)	<0.001
Creatinine (µmol/L) (n=331)				
Level on admission (µmol/L)	89 (43–626)	94 (44–505)	83 (43–626)	<0.001
Elevated on admission (n, %)	124 (38%)	91 (43%)	33 (28%)	0.005
Maximum during hospitalisation (µmol/L)	112 (48–981)	124 (51–981)	100 (48–942)	<0.001
Elevated during hospitalisation (n, %)	221 (68%)	162 (76%)	59 (53%)	<0.001

Categorical variables are described with numbers and percentages and differences between patients with type A and type B aortic dissection were analysed with χ^2^ test. Continuous variables are described with medians and ranges (min–max) and differences between patients with type A and type B aortic dissection were analysed using Mann-Whitney U test. A p value below 0.05 was considered statistically significant.

CRP, C reactive protein.

**Figure 3:**
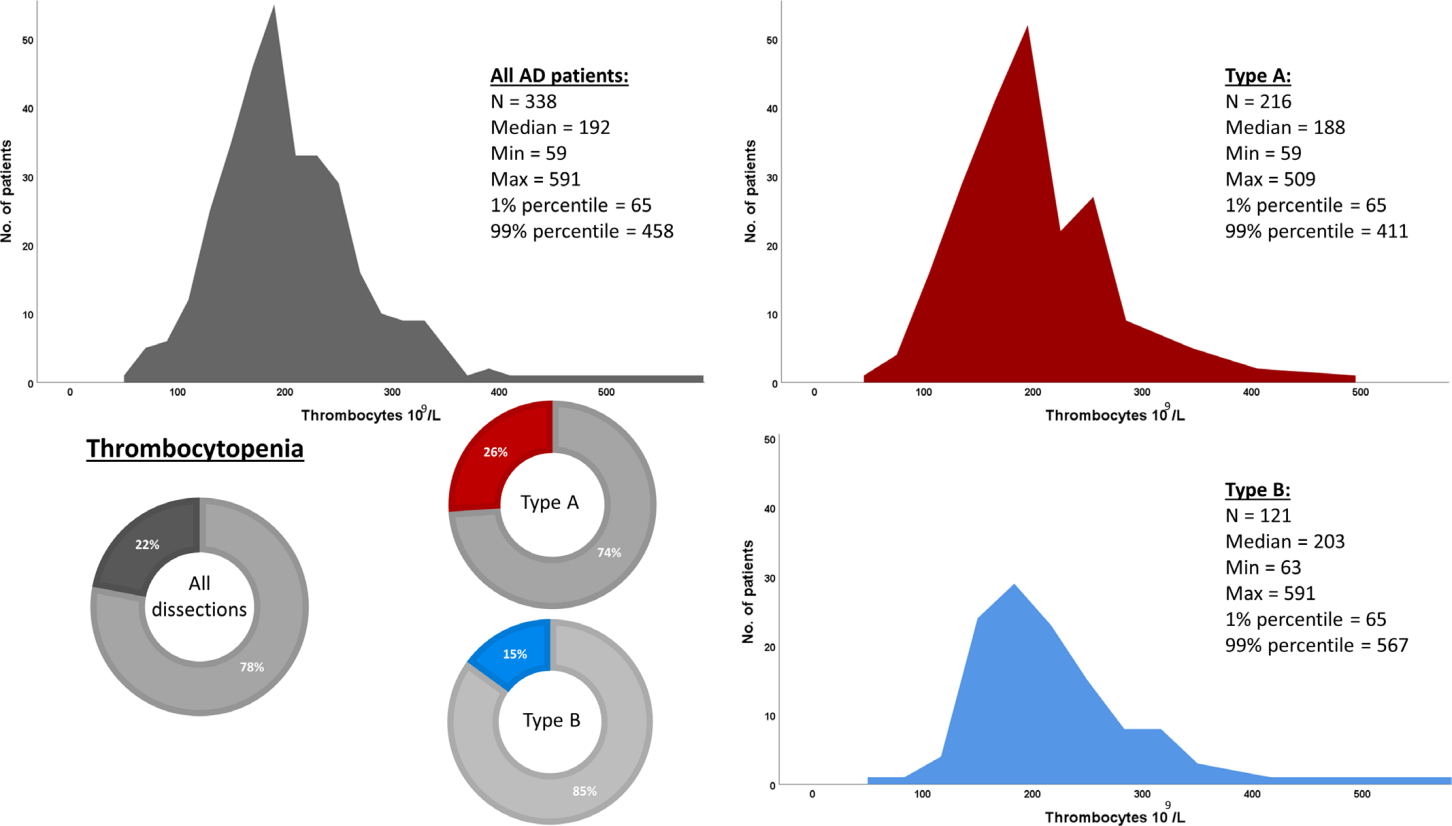
Distribution of platelet count on admission described with histograms. The proportions of patients with thrombocytopenia, defined as <145×10^9^/L for men and <165×10^9^/L for women, are depicted with circle diagrams, with colouring denoting thrombocytopenia. Results are presented for all patients and in patients with type A and type B dissection separately. AD, aortic dissection.

Platelet count was available in all but seven patients. Median level of platelets was 192×10^9^ /L. Thrombocytopenia (platelet count <165×10^9^ /L in women and <145×10^9^ /L in men) on admission was more frequent in TAD than in TBD (26% vs 15%, p=0.010) (table 3, figure 3).

### Acute management of TAD

The majority of the patients with TAD underwent OSR, 79% (n=174/220). Patients undergoing OSR were younger than patients only receiving medical therapy (median age 62 years vs 76 years, p<0.001). More women were managed non-operatively than men (29% vs 17%, p=0.033). Seven patients (4%) were treated with complementary TEVAR after OSR for TAD. Of the 46 patients managed non-operatively, 20 (43%) were denied repair because of advanced age and/or extensive comorbidity. One patient opted out from OSR. The remaining 25 patients included 10 patients presenting with cardiac arrest, 10 patients with persistent unconsciousness and 5 patients with other severe neurological deficits.

### Acute management of TBD

Of the patients with TBD, 38% (n=47/124) had complicated dissection in the acute phase. Almost half of these patients had malperfusion (47%, n=22/47) and 30% (n=14/47) had rupture/impending rupture as the predominant feature. Among patients with acute complicated TBD, 72% (n=34/47) were treated by means of surgical repair in the acute phase, 32 patients with TEVAR, 1 patient with abdominal EVAR and one patient with open thoracoabdominal aortic repair.

### Short-term mortality

Overall, 30-day mortality was 22% (n=75/344). Mortality was higher in TAD than in TBD (28% vs 11%, p<0.001). An association to higher 30-day mortality was demonstrated in patients with painless dissection compared with painful dissection (52% mortality vs 17% mortality; OR 4.30, 95% CI 1.80 to 10.28, p=0.001) and in patients with elevated admission troponin T level compared with normal levels (35% mortality with elevated troponin T vs 12% mortality with normal troponin T; OR 3.78, 95% CI 2.01 to 7.12, p <0.001), respectively.

#### Short-term mortality in TAD

In surgically managed patients, 30-day mortality was 13% (n=22/174), whereas it was 85% (n=39/46) in patients denied surgery. Among non-surgically managed patients, 46% (n=21/46) died within 24 hours, 65% (n=30/46) within 72 hours and 78% (n=36/46) within 1 week from onset of symptoms. Factors associated with higher early mortality both in TAD patients overall and in TAD patients undergoing OSR, respectively, were elevated troponin T and thrombocytopenia (table 4A). An association between painless dissection and 30-day mortality was demonstrated in TAD patients, whereas, in subgroup analysis, no such association was found in TAD patients undergoing OSR (table 4A).

**Table 4:** Factors associated with 30-day mortality in patients with type A aortic dissection (A) and type B aortic dissection (B), respectively

(A) variable	Deaths within 30 days n (%)	Crude results OR (95% CI)	Adjusted results OR (95% CI)
Type A (n=220)	61 (28%)		
Age		1.07 (1.04 to 1.10)	1.06 (1.03 to 1.09)
Men (n=144)	33 (23%)	1	1
Women (n=76)	28 (37%)	1.96 (1.07 to 3.60)	1.42 (0.75 to 2.71)
Painful dissection (n=180)	39 (22%)	1	1
Painless dissection (n=40)	22 (55%)	4.42 (2.16 to 9.05)	4.14 (1.92 to 8.93)
Normal troponin T (n=102)	17 (17%)	1	1
Elevated troponin T (n=81)	31 (38%)	3.10 (1.56 to 6.16)	2.88 (1.40 to 5.94)
Normal platelet count (n=161)	34 (21%)	1	1
Thrombocytopenia (n=56)	25 (45%)	3.01 (1.44 to 5.27)	3.09 (1.53 to 6.21)
Open surgical repair (n=174)	22 (13%)	1	1
No surgery (n=46)	39 (85%)	38.5 (15.3 to 96.6)	39.6 (13.7 to 115)
Open surgical repair (n=174)			
Age		1.01 (0.97 to 1.05)	1.01 (0.96 to 1.04)
Men (n=120)	13 (11%)	1	1
Women (n=54)	9 (17%)	1.65 (0.66 to 4.12)	1.63 (0.63 to 4.26)
Painful dissection (n=154)	17 (11%)	1	1
Painless dissection (n=20)	5 (25%)	2.69 (0.87 to 8.32)	2.65 (0.85 to 8.27)
Normal troponin T (n=90)	8 (9%)	1	1
Elevated troponin T (n=58)	12 (21%)	2.67 (1.02 to 7.02)	2.81 (1.06 to 7.49)
Normal platelet count (n=131)	11 (8%)	1	1
Thrombocytopenia (n=42)	11 (26%)	3.87 (1.56 to 9.76)	4.23 (1.63 to 10.9)

(B) Variable	Deaths within 30 days n (%)	Crude results OR (95% CI)	Adjusted results OR (95% CI)
Type B (n=124)	14 (11%)		
Age		1.06 (1.01 to 1.12)	1.07 (1.01 to 1.13)
Men (n=82)	9 (11%)	1	1
Women (n=42)	5 (12%)	1.01 (0.34 to 3.51)	1.42 (0.75 to 2.71)
Painful dissection (n=116)	11 (10%)	1	1
Painless dissection (n=8)	3 (38%)	5.73 (1.20 to 27.3)	6.69 (1.29 to 34.6)
Normal troponin T (n=73)	3 (4%)	1	1
Elevated troponin T (n=19)	4 (21%)	6.22 (1.26 to 30.7)	5.18 (1.01 to 26.5)
Normal platelet count (n=103)	14 (14%)	–	–
Thrombocytopenia (n=18)	0	–	–
Acute complicated (n=47)	14 (30%)	–	–
Acute uncomplicated (n=77)	0	–	–
Acute complicated type B treated with TEVAR* (n=32)	6 (19%)		
Age		1.03 (0.93 to 1.08)	1.00 (0.93 to 1.07)
Men (n=26)	4 (15%)	1	1
Women (n=6)	2 (33%)	2.75 (0.37 to 20.4)	2.85 (0.36 to 22.8)
Painful dissection (n=31)	5 (16%)	–	–
Painless dissection (n=1)	1 (100%)	–	–
Normal troponin T (n=18)	1 (6%)	1	1
Elevated troponin T (n=6)	3 (50%)	17.0 (1.30 to 223)	15.9 (1.20 to 212)
Normal platelet count (n=24)	6 (25%)	–	–
Thrombocytopenia (n=7)	0	–	–

Associations with 30-day death were analysed with multivariable logistic regression models, adjusting for sex and age (as a continuous variable). Results are presented for all patients with type A dissection and for patients treated with open surgical repair (A), respectively, as well as for all patients with type B dissection and patients with acute complicated type B dissection treated with TEVAR during the acute phase (B), respectively.

TEVAR, Thoracic EndoVascular Aortic Repair.

#### Short-term mortality in TBD

In acute complicated TBD, 30-day mortality was 30% (n=14/47), whereas none of the patients with acute uncomplicated TBD died within 30 days. In patients treated with TEVAR in the acute phase, 30-day mortality was 19% (n=6/32).

Painless dissection as well as elevated troponin T level on admission was associated with higher 30-day mortality in patients with TBD (table 4B). In subgroup analysis of patients with acute complicated TBD treated with TEVAR, elevated troponin T was found to be associated with higher 30-day mortality, whereas painless dissection was not (table 4B).

### Mid-term mortality

One-year mortality was 24% (n=82/344) and 3-year mortality was 28% (n=97/344). Mortality at 1 year was higher in patients with TAD than in patients with TBD (29% vs 15%, OR 3.16; 95% CI 1.71 to 5.85, p<0.001), but mid-term mortality did not differ between TAD and TBD patients, HR 1.40 (95% CI 0.97 to 2.03; p=0.072).

## Discussion

This population-based study of patients with acute AD has highlighted several important aspects of the potential prognostic roles of clinical presentation and of biochemical markers, respectively. The annual incidence was 4.1 per 100 000, two times as high in men than in women. Painless dissection as well as elevated troponin T plasma level and thrombocytopenia on admission was more commonly found in patients with TAD than in TBD. Irrespective of dissection type, both painless dissection and admission troponin T elevation, respectively, were associated with increased early mortality. Thrombocytopenia was found to be associated with adverse outcome in TAD patients, but not in TBD.

The 2014 ESC guidelines on thoracic aortic disease stated that up-to-date data on AD epidemiology are scarce.^6^ Accordingly, the true incidence of AD has been hard to determine.^6^ In the Oxford vascular study (n=54 acute AD cases), the annual incidence was 6 per 100 000, in a population-based setting in a county of 93 000 inhabitants.^16^ More recent population-based studies from Iceland, Canada and Sweden reported an annual incidence of 2.5, 4.6 and 7.1 per 100 000, respectively.^2 17 18^ The present study focused on assessing the annual incidence of acute spontaneous AD, that is, non-traumatic and non-iatrogenic, in the adult population (≥18 years of age). All hospitalised patients were identified with high certainty, but data on potential out-of-hospital deaths from acute AD were not available. The men/women ratio of 2:1 reported in this study is higher than in some of the previous reports that indicated a ratio of 1.5:1.^16 18^

Painless acute AD has been described as an uncommon but highly lethal condition, more frequently occurring in patients with TAD than TBD.^9 19 20^ The proportion with painless dissection, 14%, in our report was higher than described by IRAD.^7 9 20^ Presumably, this population-based study reflects the true proportions of patients with painless dissection more accurately than potentially selective register data, as patients with painless dissection, who more often present with worse clinical status, may not have been transferred to IRAD centres.^7^ Our data indicated that, in agreement with prior findings, painless dissection was more common in TAD than in TBD.^9^ Similar to earlier reports, neurological deficit was the predominant symptom among these patients.^19^ There was an association between painless dissection and increased early mortality, both in patients with TAD and in patients with TBD, a finding that confirmed the worse prognosis related to atypical presentation.^7 20^

The role of biochemical markers in the management of patients with acute AD is poorly understood. A meta-analysis from 2016 reported that 27% of acute AD patients had elevated troponin, which was associated with increased early mortality.^13^ The report did not differ between patients with TAD and TBD, however.^13^ In the present study, 36% had elevated troponin T on admission, more commonly seen in TAD than in TBD. It was associated with higher 30-day mortality, both in TAD and in TBD patients, respectively. That association might be explained by delayed diagnosis, co-occurring myocardial ischaemia or a combination of the two, as the primary suspicion in case of troponin elevation would most probably be acute coronary syndrome.^21^ Other conditions that may result in troponin elevation include cardiac failure, hypotension/shock and renal failure, all being possible reasons also for troponin elevation in patients with simultaneous acute AD.^22 23^ Although AD has been suggested as a possible differential diagnosis in patients with elevated troponin T, the mechanism by which AD results in release of the cardiac-specific biomarker has not been fully explained.^23^ The cut-off level of 14 ng/L is assay-specific and based on the 99% percentile in healthy individuals, as recommended by the ESC guidelines for the management of acute coronary syndrome.^23^ It seems reasonable to use the same cut-off level when managing patients with AD but that has not been further elucidated. Still, even at the 80th percentile in this cohort of acute AD patients, troponin T was 27 ng/L, thus not very markedly elevated.

The role of platelet count and of thrombocytopenia in patients with AD is poorly studied. A high mean platelet volume/platelet count ratio has been associated with increased mortality in patients with TAD.^24^ In this study, thrombocytopenia on admission was found also to be associated with early mortality in patients with TAD. Even mild preoperative thrombocytopenia (platelet count 100–150×10^9^/L) has been associated with adverse events and increased early mortality after isolated coronary artery bypass grafting.^25^ Hence, it is plausible that the light platelet count reduction observed in most of our acute AD patients with thrombocytopenia was indeed clinically significant.

A couple of risk calculators and risk stratification tools with different purposes have been suggested for patients with acute AD. In patients with TBD, prediction models based on a combination of morphological markers and clinical presentation, including refractory pain and refractory hypertension, were developed to identify patients with high risk of suffering late complications.^26 27^ The Penn classification, a tool to predict early mortality, is based on clinical presentation alone.^28^ A more diverse risk scoring system could presumably help clinicians in several ways, such as in identifying patients unfit for surgery. The Glasgow Aneurysm Score was developed to identify patients with ruptured abdominal aortic aneurysm with particularly high risk of peri-/postoperative mortality.^29^ In AD patients, such a scoring system should ideally identify patients with high complication risk and potential benefit from longer stay in the ICU, longer hospital stay and a tighter follow-up regimen. At best, it could also be used as a prognostic instrument in communication with patients and relatives. Based on our findings, the scoring system should preferably include information on painless onset of dissection, plasma level of troponin T and platelet count, respectively, on admission.

This study is limited by its retrospective design. Data including symptoms, concurrent diseases, clinical findings, etc were registered based on medical records, not necessarily providing a full description. The decision to analyse certain biochemical markers was made in the emergency room by the treating physician, introducing possible selection bias. In contrast to the Oxford vascular study and the national Swedish study, patients deceased outside of hospital were not included in this study. However, the study was based on thorough retrospective review of data from hospital records, that is, not register-based, ascertaining that only true acute spontaneous AD cases were included. The most important strength of this study is the population-based design, including all patients hospitalised with acute AD in the catchment area during the study period with complete follow-up of the patients, providing knowledge of all patients admitted for acute AD, not only patients undergoing repair.

## Conclusion

In summary, this population-based study demonstrated unique insight into the incidence of acute AD and the roles of biochemical markers and the importance of the mode of clinical presentation, respectively. The incidence of acute AD was 4.1 per 100 000, higher in men than in women. Painless dissection and elevated plasma troponin T levels on admission were both associated with higher 30-day mortality, both in patients with TAD and TBD, respectively, whereas thrombocytopenia was associated with higher early mortality only in TAD patients. These determinants may become useful tools in early risk stratification and aid in future decision-making.

## Data Availability

Data are available upon reasonable request. Deidentified participant data. Contact details: orc-id 0000-0003-1326-3945.

## References

[1] Rogers AM , Hermann LK , Booher AM , et al . Sensitivity of the aortic dissection detection risk score, a novel guideline-based tool for identification of acute aortic dissection at initial presentation: results from the international registry of acute aortic dissection. Circulation 2011;123:2213–8. 10.1161/CIRCULATIONAHA.110.988568 21555704

[2] Smedberg C , Steuer J , Leander K , et al . Sex differences and temporal trends in aortic dissection: a population-based study of incidence, treatment strategies, and outcome in Swedish patients during 15 years. Eur Heart J 2020;41:2430–8. 10.1093/eurheartj/ehaa446 32558879 PMC7340356

[3] Daily PO , Trueblood HW , Stinson EB , et al . Management of acute aortic dissections. Ann Thorac Surg 1970;10:237–47. 10.1016/s0003-4975(10)65594-4 5458238

[4] Hirst AE , Johns VJ , Kime SW . Dissecting aneurysm of the aorta: a review of 505 cases. Medicine (Baltimore) 1958;37:217–79. 10.1097/00005792-195809000-00003 13577293

[5] Suzuki T , Mehta RH , Ince H , et al . Clinical profiles and outcomes of acute type B aortic dissection in the current era: lessons from the International Registry of Aortic Dissection (IRAD). Circulation 2003;108 Suppl 1:II312–7. 10.1161/01.cir.0000087386.07204.09 12970252

[6] Erbel R , Aboyans V , Boileau C , et al . ESC guidelines on the diagnosis and treatment of aortic diseases: document covering acute and chronic aortic diseases of the thoracic and abdominal aorta of the adult. The task force for the diagnosis and treatment of aortic diseases of the European Society of Cardiology (ESC). Eur Heart J 2014;35:2873–926. 10.1093/eurheartj/ehu281 25173340

[7] Evangelista A , Isselbacher EM , Bossone E , et al . Insights from the international registry of acute aortic dissection: a 20-year experience of collaborative clinical research. Circulation 2018;137:1846–60. 10.1161/CIRCULATIONAHA.117.031264 29685932

[8] Hagan PG , Nienaber CA , Isselbacher EM , et al . The international registry of acute aortic dissection (IRAD): new insights into an old disease. JAMA 2000;283:897–903. 10.1001/jama.283.7.897 10685714

[9] Park SW , Hutchison S , Mehta RH , et al . Association of painless acute aortic dissection with increased mortality. Mayo Clin Proc 2004;79:1252–7. 10.4065/79.10.1252 15473405

[10] Upchurch GR , Nienaber C , Fattori R , et al . Acute aortic dissection presenting with primarily abdominal pain: a rare manifestation of a deadly disease. Ann Vasc Surg 2005;19:367–73. 10.1007/s10016-004-0171-x 15735946

[11] Ranasinghe AM , Bonser RS . Biomarkers in acute aortic dissection and other aortic syndromes. J Am Coll Cardiol 2010;56:1535–41. 10.1016/j.jacc.2010.01.076 21029872

[12] Suzuki T , Distante A , Zizza A , et al . Diagnosis of acute aortic dissection by D-Dimer: the international registry of acute aortic dissection substudy on biomarkers (IRAD-bio) experience. Circulation 2009;119:2702–7. 10.1161/CIRCULATIONAHA.108.833004 19433758

[13] Vrsalovic M . Prognostic effect of cardiac troponin elevation in acute aortic dissection: a meta-analysis. Int J Cardiol 2016;214:277–8. 10.1016/j.ijcard.2016.03.230 27082771

[14] Xie E , Liu J , Liu Y , et al . Association between platelet counts and morbidity and mortality after endovascular repair for type B aortic dissection. Platelets 2022;33:73–81. 10.1080/09537104.2020.1847266 33213236

[15] Elm E von , Altman DG , Egger M , et al . The strengthening the reporting of observational studies in epidemiology (STROBE) statement: guidelines for reporting observational studies. BMJ 2007;335:806–8. 10.1136/bmj.39335.541782.AD 17947786 PMC2034723

[16] Howard DPJ , Banerjee A , Fairhead JF , et al . Population-based study of incidence and outcome of acute aortic dissection and premorbid risk factor control: 10-year results from the oxford vascular study. Circulation 2013;127:2031–7. 10.1161/CIRCULATIONAHA.112.000483 23599348 PMC6016737

[17] McClure RS , Brogly SB , Lajkosz K , et al . Epidemiology and management of thoracic aortic dissections and thoracic aortic aneurysms in Ontario, Canada: a population-based study. J Thorac Cardiovasc Surg 2018;155:2254–64. 10.1016/j.jtcvs.2017.11.105 29499864

[18] Melvinsdottir IH , Lund SH , Agnarsson BA , et al . The incidence and mortality of acute thoracic aortic dissection: results from a whole nation study. Eur J Cardiothorac Surg 2016;50:1111–7. 10.1093/ejcts/ezw235 27334108

[19] Marroush TS , Boshara AR , Parvataneni KC , et al . Painless aortic dissection. Am J Med Sci 2017;354:513–20. 10.1016/j.amjms.2016.11.005 29173364

[20] Tolenaar JL , Hutchison SJ , Montgomery D , et al . Painless type B aortic dissection: insights from the international registry of acute aortic dissection. Aorta (Stamford) 2013;1:96–101. 10.12945/j.aorta.2013.13-014 26798680 PMC4682707

[21] Rapezzi C , Longhi S , Graziosi M , et al . Risk factors for diagnostic delay in acute aortic dissection. Am J Cardiol 2008;102:1399–406. 10.1016/j.amjcard.2008.07.013 18993163

[22] Suzuki T , Lyon A , Saggar R , et al . Editor’s choice-biomarkers of acute cardiovascular and pulmonary diseases. Eur Heart J Acute Cardiovasc Care 2016;5:416–33. 10.1177/2048872616652309 27221957

[23] Roffi M , Patrono C , Collet J-P , et al . ESC guidelines for the management of acute coronary syndromes in patients presenting without persistent ST-segment elevation: task force for the management of acute coronary syndromes in patients presenting without persistent ST-segment elevation of the European Society of Cardiology (ESC). Eur Heart J 2016;37:267–315. 10.1093/eurheartj/ehv320 26320110

[24] Li D-Z , Chen Q-J , Sun H-P , et al . Mean platelet volume to platelet count ratio predicts in-hospital complications and long-term mortality in type A acute aortic dissection. Blood Coagul Fibrinolysis 2016;27:653–9. 10.1097/MBC.0000000000000449 26575495

[25] Nammas W , Dalén M , Rosato S , et al . Impact of preoperative thrombocytopenia on the outcome after coronary artery bypass grafting. Platelets 2019;30:480–6. 10.1080/09537104.2018.1466389 29676943

[26] Sailer AM , van Kuijk SMJ , Nelemans PJ , et al . Computed tomography imaging features in acute uncomplicated Stanford type-B aortic dissection predict late adverse events. Circ Cardiovasc Imaging 2017;10:e005709. 10.1161/CIRCIMAGING.116.005709 28360261 PMC5413355

[27] Lombardi JV , Hughes GC , Appoo JJ , et al . Society for Vascular Surgery (SVS) and Society of Thoracic Surgeons (STS) reporting standards for type B aortic dissections. J Vasc Surg 2020;71:723–47. 10.1016/j.jvs.2019.11.013 32001058

[28] Olsson C , Hillebrant C-G , Liska J , et al . Mortality in acute type A aortic dissection: validation of the penn classification. Ann Thorac Surg 2011;92:1376–82. 10.1016/j.athoracsur.2011.05.011 21855849

[29] Özen A , Unal EU , Mola S , et al . Glasgow aneurysm score in predicting outcome after ruptured abdominal aortic aneurysm. Vascular 2015;23:120–3. 10.1177/1708538114533539 24841850

