# Supplementary material for: Incidence, presentation and outcome of acute aortic dissection: results from a population-based study
Source: Open Heart. 2024 Mar 13;11(1):e002595. doi: 10.1136/openhrt-2023-002595 (PMC10941176; doi:10.1136/openhrt-2023-002595)
Supplement: Supplementary data [file openhrt-2023-002595supp001.pdf]

Supplement table 1

Biochemical markers presented with cut-off levels and assays.

Variable	Unit	Cut-off level	Method	Company
**Troponin T**	ng/L	>14 ng/L	Electrochemiluminescence immunoassay	Cobas®, Roche Diagnostics
**Platelet count**	x 10^9	<145 x 10^9/L in men, <165 x 10^9/L in women	Flow cytometry or manual count	-
**D-dimer**	mg/L	>0.25 mg/L	Immunoturbidimetric assay	Tina-quant®, Roche Diagnostics
**CRP**	mg/L	>3 mg/L	Immunoturbidimetric assay	Cobas®, Roche Diagnostics
**Creatinine**	**µmol/L**	>100 µmol/L in men, >90 µmol/L in women	Jaffe or enzymatic method	Beckman Coulter®, Cobas®, Roche Diagnostics
