# Supplementary figures and images for: Incidence, presentation and outcome of acute aortic dissection: results from a population-based study
Source: Open Heart. 2024 Mar 13;11(1):e002595. doi: 10.1136/openhrt-2023-002595 (PMC10941176; doi:10.1136/openhrt-2023-002595)

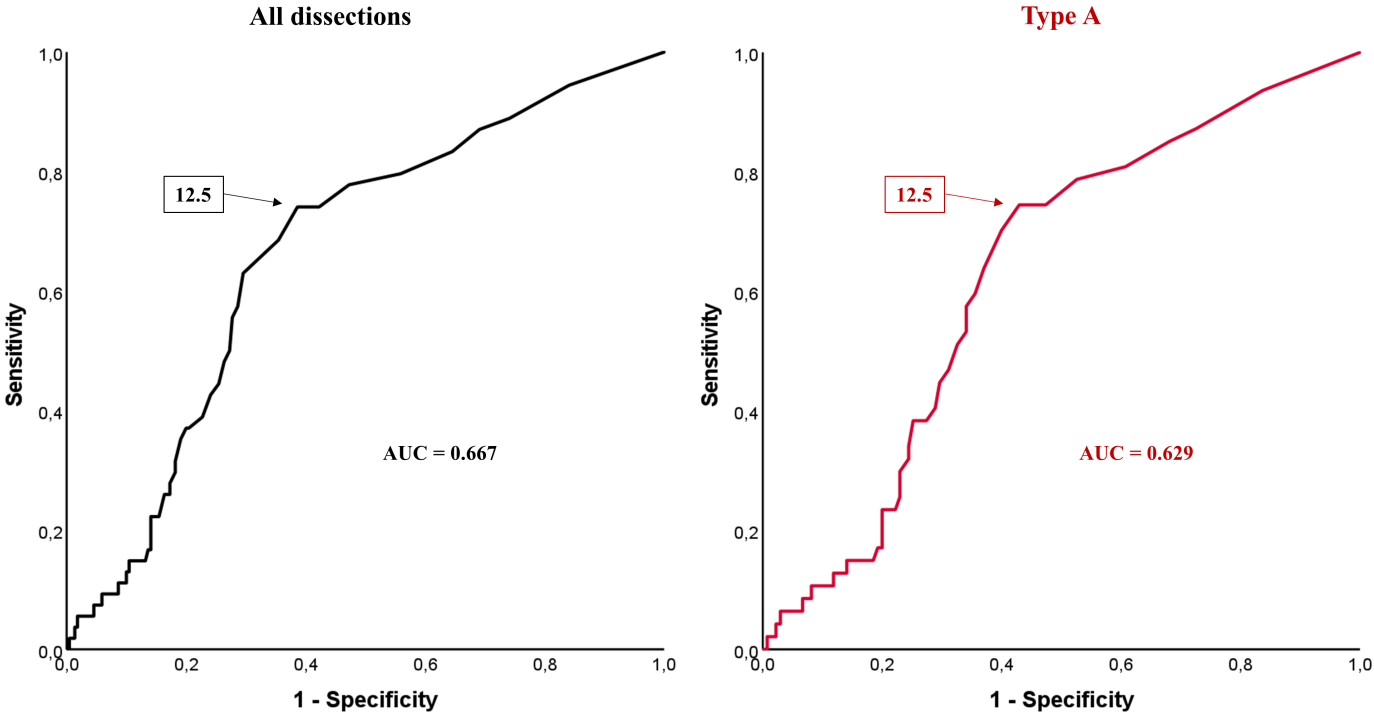

Supplement: Supplementary data [file openhrt-2023-002595supp002.pdf]
