# Supplementary material for: Incidence, presentation and outcome of acute aortic dissection: results from a population-based study
Source: Open Heart. 2024 Mar 13;11(1):e002595. doi: 10.1136/openhrt-2023-002595 (PMC10941176; doi:10.1136/openhrt-2023-002595)
Supplement: Supplementary data [file openhrt-2023-002595supp003.pdf]

Supplement figure 1

Receiver operating characteristic (ROC) curves for level of troponin T and 30-day mortality for (a) all patients and (b) for patients with TAD, respectively. Areas under the curve (AUC) as well as best discriminatory values are presented.
